# Hyperkalemic Emergency: When You Have Taken a Few Too Many KCl Tablets

**DOI:** 10.7759/cureus.10499

**Published:** 2020-09-17

**Authors:** Mahmoud Ibrahim, Christina Seto, Tracy MacIntosh

**Affiliations:** 1 Internal Medicine, University of Central Florida College of Medicine/Hospital Corporation of America Graduate Medical Education Consortium, Kissimmee, USA; 2 Medicine, University of Central Florida College of Medicine, Orlando, USA; 3 Emergency Medicine, Osceola Regional Medical Center, Kissimmee, USA

**Keywords:** hyperkalemia, hyperkalemic emergency, potassium, critical care, emergency medicine, internal medicine, ekg, hemodialysis, insulin, bipolar disorder

## Abstract

Hyperkalemia is a common clinical problem that varies significantly in severity and indications for treatment. Hyperkalemic emergency exists when there are clinical signs or symptoms, including cardiac conduction abnormalities. The combination of nebulized albuterol and insulin with glucose is most effective for managing clinically significant hyperkalemia. Prompt recognition of hyperkalemic emergency, immediate interventions to lower extracellular potassium, and involvement of multiple disciplines (including critical care and nephrology) are essential to addressing this life-threatening presentation.

## Introduction

Hyperkalemia is a common clinical problem that presents in both the emergency and inpatient medicine settings. The frequency of hyperkalemia in the United States is reported to be between 2.6% and 3.2% in the general population [1]. Potassium is one of the most abundant cations in body [2], and disturbances to its homeostasis can have serious and even fatal consequences. Several factors can precipitate the formation of hyperkalemia, such as poor renal function, the use of certain medications, and independent demographic features [3,4]. The severity of hyperkalemia drives its management, which may be primarily through medical or procedural techniques, or through a combination of the two [1-15]. In this case report, we present a case of severe hyperkalemia due to combination of medication and supplement overdose, which required emergent hemodialysis due to hemodynamic instability.

## Case presentation

A 48-year-old female with a history of bipolar disorder, hypertension, and gastro-esophageal reflux disease (GERD) presented to the emergency department stating, “I have low potassium.” She reported having hypokalemia for the past 10 years and had been taking 60 mEq daily of supplemental potassium chloride (KCl). She began having muscle cramps one day prior to presentation and decided to “double up” on her KCl tablets, thinking her symptoms were due to hypokalemia. She denied any recent depressive symptoms or suicidal ideation. She had two days of non-bloody, non-bilious vomiting associated with multiple episodes of non-bloody diarrhea. She denied fevers, chills, or night sweats.

On presentation to the emergency department, vital signs were temperature 97.1 degrees Fahrenheit, heart rate 52 beats per minute, blood pressure 135/74 mmHg, and respiratory rate 17 breaths per minute. On physical examination, she was awake, alert, and oriented. She had a normal physical exam including focused cardiovascular and neurological examinations. The most remarkable abnormal laboratory results were serum potassium (K) 9.6 mmol/L, serum chloride (Cl) 117 mmol/L, blood urea nitrogen (BUN) 73 mg/dL, and creatinine 4.67 mg/dL (with no known baseline). White blood cell (WBC) count was elevated at 13.28 K/mm³ with normal differential. All laboratory values are shown in Table 1. 

**Table 1:** Initial Laboratory Results

Laboratory Test	Patient’s Result	Reference Range
White blood cell count (WBC)	13.28 K/mm^3^	4.0-12.0 K/mm^3^
Hemoglobin (Hb)	12.5 g/dL	12.0-16.0 g/dL
Platelet count (Plt)	306 K/mm^3^	130-400 K/mm^3^
Sodium (Na)	136 mmol/L	136-145 mmol/L
Potassium (K)	9.6 mmol/L	3.7-5.1 mmol/L
Chloride (Cl)	117 mmol/L	98-107 mmol/L
Bicarbonate (CO_2_)	16 mmol/L	21-32 mmol/L
Blood urea nitrogen (BUN)	73 mg/dL	7-18 mg/dL
Creatinine	4.67 mg/dL	0.55-1.3 mg/dL
Glucose	101 mg/dL	74-106 mg/dL
Calcium	9.4 mg/dL	8.4-10.1 mg/dL
Phosphorus	3.3 mg/dL	2.5-4.9 mg/dL

**Figure 1:**
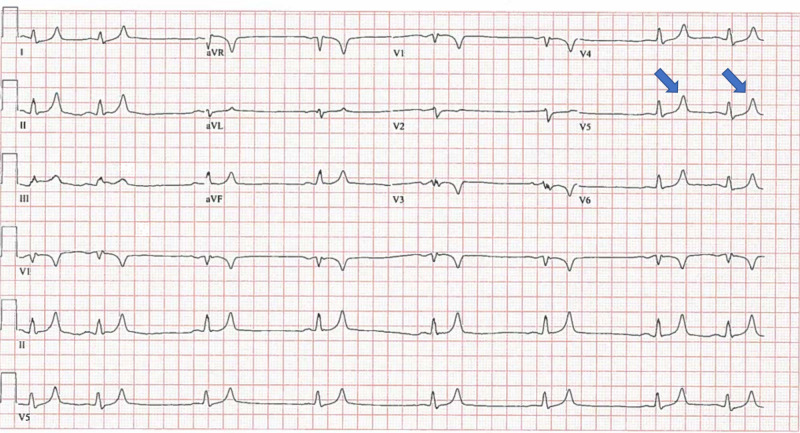
Initial Electrocardiogram (ECG) This is the initial ECG upon the patient's arrival to the emergency department. It shows normal sinus rhythm with frequent premature ventricular contractions in the pattern of bigeminy. Peaked T waves are labeled with arrows.

**Figure 2:**
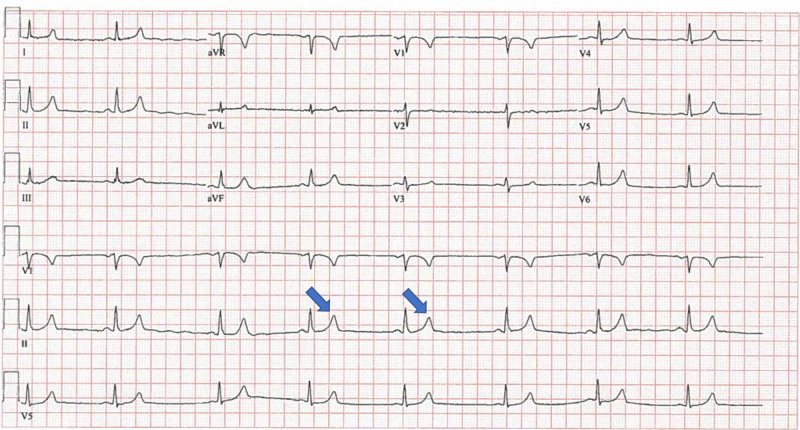
Repeat Electrocardiogram (ECG) This ECG shows sinus bradycardia with heart rate of 47 beats per minute. Once again, peaked T waves are labeled with arrows.

Initial electrocardiogram (ECG) showed normal sinus rhythm with frequent premature ventricular contractions in a pattern of bigeminy (Figure 1). Repeat electrocardiogram showed sinus bradycardia with heart rate (HR) 47 bpm and peaked T waves (Figure 2).

The patient received treatment with 50 mEq IV sodium bicarbonate, 1 gram IV calcium gluconate, five units IV insulin with one ampule of D50, and polysterene sulfate 60 grams orally. Shortly after, she became progressively bradycardic and hypotensive. An emergent trialysis catheter was placed, and she was started on a norepinephrine drip and began emergent hemodialysis. 

Her serum potassium normalized after hemodialysis to 4.2 mmol/L and her creatinine improved to 1.29 mg/dL at discharge. The patient was evaluated by the psychiatry team due to concerns for self-harm; however, the patient denied, and her ingestion was ultimately determined to be a therapeutic misadventure.

## Discussion

As a primarily intracellular cation, potassium has an inherent concentration gradient between its intracellular and extracellular components [1,2,13,14]. This concentration gradient is the basis of the cellular resting membrane potential, and as such, potassium homeostasis is the basis for cellular integrity. Hyperkalemia is defined as a serum potassium >5.0 mmol/L, and can be the result of increased potassium absorption, decreased potassium excretion, or a shift of potassium across the cell membrane [2,14]. Hyperkalemia contributes to cardiac arrhythmias by destabilizing the myocardial conduction system [12]. Decreased resting membrane potential in the setting of hyperkalemia leads to increased cardiac depolarization, myocardial excitability, and cardiac instability, with subsequent arrhythmia formation including ventricular fibrillation and asystole. Tall, peaked T waves are typically the earliest manifestations of hyperkalemia and occur before changes to the QRS complex. In the absence of any other ECG findings, peaked T waves are rarely associated with life-threatening arrhythmias. QRS complex changes (uniform widening) are often evident with increasingly severe hyperkalemia. Loss of P waves then follows, ultimately leading to the formation of sine waves - the fusion of wide QRS complexes with ST-T segments. Additionally, severe hyperkalemia can manifest with sinus bradycardia or arrest, atrioventricular (AV) blocks, and loss of pacemaker capture. Hyperkalemia can impair conduction in the His-Purkinje system and can also cause fascicular and bundle branch blocks.

IV insulin, often along with one ampule of D50 to avoid hypoglycemia, is given to lower the serum potassium concentration by driving potassium intracellularly and enhancing the activity of the Na/K ATPase pump, thereby promoting intracellular potassium shifts, taking effect within 10-15 minutes. Insulin should not be given to patients who have concurrent basal or long-acting insulin, including subcutaneous infusion pumps. Albuterol 10-15 mg nebulized is also used to transfer potassium intracellularly, with onset of 60-90 minutes. The combinatiosn of insulin/glucose and nebulized albuterol are likely most effective for lowering potassium emergently [16-18].

Calcium gluconate is commonly used in the management of life-threatening arrhythmias in the setting of hyperkalemia due to immediate onset and effects. Adult dosing is 1.5-3 g IV q2-5 minutes PRN with a maximum dosing of 200 mg/min or 3 g/episode or 15 g/day. Adverse reactions include hypercalcemia, accompanied by vasodilation, hypotension, bradycardia, syncope, and dizziness, as well as more severe symptoms such as subsequent arrhythmias and extravasation necrosis. Sodium bicarbonate also works by transcellular potassium shifting, but has limited demonstrated efficacy in lower serum K levels. Sodium polystyrene sulfonate (Kayexalate) has been used in the management of hyperkalemia as a potassium binder. Adult dosing is 15 g PO daily up to four times a day or alternatively 30-50 g PR q6h. Time of onset is typically 2-12 hours, but peak time is variable. The efficacy has not been well established, and given its lack of palatability and risks of GI obstruction, perforation, and colonic necrosis, this medication should be used with caution in the emergency setting [19,20].

Hemodialysis is often the mainstay of treatment for patients with persistent EKG changes or insufficient response to medical management, specifically in patients with renal failure [4,12]. However, hemodialysis may not be a necessary intervention for patients with normal renal function, even in the setting of extreme hyperkalemia [4]. In the rare event of potassium overdose, procedural techniques may be necessary to remove a conglomeration of pills that have formed a pharmacobezoar [5,7,9,10,15]. Finally, patiromer is a newer medication that binds potassium in the GI tract and increases fecal excretion. It is currently indicated in patients with CKD 3 and 4 or patients taking potassium-sparing diuretics or renin-angiotensin-aldosterone system inhibitors, but not for emergent management of hyperkalemia due to its delayed onset of action.

Emergent treatment of true hyperkalemia focuses on stabilizing the membranes, shifting potassium into the cells, and removing potassium from the body [3,12]. Immediate management includes calcium gluconate to stabilize the cell membrane in the setting of EKG changes, followed by insulin or albuterol to promote intracellular shifts of potassium [2,3]. Hemodialysis may be indicated to remove excess potassium from the body [2,3,13]. Novel potassium-binding agents may be effective in managing hyperkalemia in the non-emergent setting [1,3,12,13].

## Conclusions

Hyperkalemia is a common clinical problem that varies significantly in severity and indications for treatment. Hyperkalemic emergency exists when there are clinical signs or symptoms including conduction abnormalities or muscle paralysis. The combination of nebulized albuterol and insulin with glucose is most effective for managing clinically significant hyperkalemia. Intravenous calcium will only stabilize the cardiac membrane for around 30-60 minutes and is a transient measure that should be used as an adjunct with definitive serum potassium-lowering treatments. Prompt recognition of hyperkalemic emergency and involvement of multiple disciplines (including critical care and nephrology) is essential to addressing this life-threatening presentation.
